# From Genome Variation to Molecular Mechanisms: What we Have Learned From Yeast Mitochondrial Genomes?

**DOI:** 10.3389/fmicb.2022.806575

**Published:** 2022-01-20

**Authors:** Weilong Hao

**Affiliations:** Department of Biological Sciences, Wayne State University, Detroit, MI, United States

**Keywords:** mutation, recombination, repeat, mobile introns, gene conversion, GC-content

## Abstract

Analysis of genome variation provides insights into mechanisms in genome evolution. This is increasingly appreciated with the rapid growth of genomic data. Mitochondrial genomes (mitogenomes) are well known to vary substantially in many genomic aspects, such as genome size, sequence context, nucleotide base composition and substitution rate. Such substantial variation makes mitogenomes an excellent model system to study the mechanisms dictating mitogenome variation. Recent sequencing efforts have not only covered a rich number of yeast species but also generated genomes from abundant strains within the same species. The rich yeast genomic data have enabled detailed investigation from genome variation into molecular mechanisms in genome evolution. This mini-review highlights some recent progresses in yeast mitogenome studies.

## Introduction

Mitochondrial genomes (mitogenomes) originated from an alpha-proteobacterium *via* endosymbiosis (Lang et al., 1999), and have adopted radically different shapes, sizes, and organizations (Burger et al., 2003; Shao et al., 2009; Sloan et al., 2012; Smith et al., 2012). The great variation of mitogenome diversity and complexity has revolutionized our view of genome evolution and facilitated development of new evolutionary theories (Bazin et al., 2006; Lynch et al., 2006; Whitney and Garland, 2010; Sloan et al., 2012; Christensen, 2013). The excitement of mitogenomes inspired more sequencing projects, and perhaps more importantly, many mindful and in-depth comparative genomics studies (Smith, 2015). These efforts continue to push the boundaries of our understanding in genome evolution.

Mitogenomes are highly variable among yeast species. Mitogenomes show substantial differences in genome size and organization, GC-content, mutation rates, and recombination frequencies. As mutation is the fundamental source of genetic variation, mitogenome differences provide important insights into the underlying mechanisms in the mutation processes. The budding yeast *Saccharomyces cerevisiae* is among the best-studied model organisms, an abundant number of mitogenomes have been sequenced for *S. cerevisiae* (Strope et al., 2015; De Chiara et al., 2020) and related species (Freel et al., 2014; Nguyen et al., 2020b). The abundant genomic data have allowed comparative analyses of mitogenomes among closely related yeast species and also intraspecific strains within a species. Detailed variations among closely related mitogenomes become uniquely informative to help identify molecular mechanisms driving the genomic changes.

## Mitochondrial DNA Deletion

The mitochondrial DNAs (mtDNAs) of related yeast strains is well known to show hypervariability in sequence [reviewed in Borst and Grivell (1978)]. Studies of hypervariable yeast mitogenomes have led to discoveries of new molecular and cellular mechanisms (Dujon, 2020). Yeast spontaneously loses mtDNAs and develops respiratory-incompetent petite colonies (rho^–^) (Whittaker et al., 1972). Petite colonies arise naturally under normal growth conditions, but the frequency of petite formation varies among strains. In *S. cerevisiae*, the frequency of developing spontaneous petite colonies varies by about 100-fold between laboratory strains versus natural isolates (Dimitrov et al., 2009). The formation of petite colonies, or petite-positive trait, has been associated with whole-genome duplication of the nuclear genome (Piskur, 2001), which provides the basis for neofunctionalization. A survey in more than one hundred strains, however, shows that the petite positive trait is throughout the Saccharomycetaceae family, much beyond the whole genome duplication species (Fekete et al., 2007). Some deletions in yeast mtDNAs are mediated by gene conversion between GC cluster repeats (Weiller et al., 1991), which are characterized by high GC content and palindromic structure (Yin et al., 1981) (also described below). Petite formation, as a genetic trait, has been studied in genome-wide association studies to identify the associated genes (Dimitrov et al., 2009). The different frequencies in petite formation among strains have been associated with genetic variation in at least four nuclear-encoded genes, the mtDNA polymerase (MIP1) (Baruffini et al., 2007a) and other less-well studied SAL1, CAT5, and MKT1 genes (Dimitrov et al., 2009).

## Movement of Mitochondrial Introns

Genetic crosses of different mitochondrial genotypes have been conducted in *S. cerevisiae* to understand yeast mitochondrial inheritance and genetics (Wilkie and Thomas, 1973). Mitochondrial markers are inherited in a non-mendelian manner (Jacquier and Dujon, 1985). A well-studied example is the omega (ω) locus in 21S rRNA (Butow, 1985), in which the ω+ strains can transfer the whole intron unidirectionally to an intron-less locus (Dujon et al., 1974). The ω locus belongs to group I intron, a class of self-catalytic ribozymes that often encode a homing endonuclease gene (HEG) (Perlman and Butow, 1989). The self-spicing intron and intron-encoded endonuclease play an important role in driving intron invasion and mobility (Dujon, 1989). The mobility and invasion of the ω intron can go beyond the species boundary. The phylogeny of ω-HEG is significantly different from that of the host 21S rRNA (Goddard and Burt, 1999), suggesting cross-species horizontal transfer of ω-HEG. Given the highly invasive nature of the ω intron, one would expect most yeast strains to be ω+. Yet, many yeast strains remain ω–. Goddard and Burt (1999) provided a framework to explain the sporadic distribution of the ω intron; after invasion, the omega-HEGs undergo rapid degeneration and loss, followed by reinvasion. This process is coined as the Goddard–Burt life cycle by Mukhopadhyay and Hausner (2021). The actual life cycle of introns is likely much more complex than cyclical invasion, degeneration, loss and then reinvasion.

In the Saccharomycetaceae family, mitochondrial introns are found in three genes, *cox1*, *cob*, and 21S rRNA, in total 17 intron positions (Wu et al., 2015). Each intron has a unique distribution pattern, and intron content often varies substantially even among small numbers of conspecific strains within species. Except the *cox1* i1 intron, all other 16 introns are sporadically distributed. The evolutionary turnover rates of gain and loss among these introns were quantitatively measured (Wu et al., 2015). The high-mobility introns documented in genetic crosses do not necessarily have faster turnover rates than low-mobility introns. The *cox1* i1 intron is currently only found in *S. cerevisiae* strains, with a high rate of intron insertion, it will not be surprising if the *cox1* i1 intron is present in some upcoming non-*S. cerevisiae* mitogenomes. Furthermore, phylogenetically mosaic sequences are evident in both introns and HEGs (Wu and Hao, 2014). Thus, intron and its encoded HEG do not always transmit together as a unit. These findings support that gene conversion between the donor and recipient sequences can take place at both the gene and intragenic levels and lead to insertion or deletion of the adjacent HEG/intron content (Wu and Hao, 2014).

## Elevated Sequence Evolution Near Mobile Introns

The distribution of mutation along DNA sequences is not uniform, and yeast mitogenomes are no exception. The recent availability of abundant yeast population genomic data makes it possible to examine genetic diversity along mitochondrial genes. One striking finding is the increased density of single nucleotide polymorphisms (SNP) in exon regions approaching intron boundaries (Repar and Warnecke, 2017). Although intron mobility is recognized to play a critical role in driving the sequence diversity of host genes, the underlying mechanisms cannot be easily identified. There are two possible mechanisms that can increase SNP density in exons (Repar and Warnecke, 2017). First, diverse exonic sequences are gene conversion tracts, or known as co-conversion tracts, acquired from distantly related species. Horizontal transfer of introns and co-conversion tracts has been well documented in plant mitogenomes (Cho and Palmer, 1999). Since most plant mitogenomes have exceptionally low mutation rates, shared long co-conversion tracts among distantly related intron-containing sequence can be easily and convincingly identified (Sanchez-Puerta et al., 2011). Similar to plant mitochondrial introns, yeast mitochondrial introns also undergo horizontal transfer (Wu et al., 2015) and gene conversion at intragenic level (Wu and Hao, 2014). The relative high sequence divergence among yeast mitogenomes makes it challenging to accurately identify the donor species of the diverse co-conversion tracts. Alternatively, the flanking regions of each intron insert site are mutation hotspots because of endonuclease activity and subsequent error-prone repair. Yeast mitogenomes contain stand-alone HEGs, which are not associate with mitochondrial introns. A strong association is evident between the presence of a stand-alone endonuclease gene and high sequence diversity at the end of the endonuclease-adjacent gene (Wu and Hao, 2019). This finding is consistent with the notion that the recognition sites of endonuclease are mutation hotspots.

## The Rebirth of GC-Clusters

The *var1* gene (or called *rps3* in non-yeast fungi) is another well-studied example of unidirectional inheritance in *S. cerevisiae* (Strausberg and Butow, 1981). The *var1* gene is polymorphic, and different forms of the *var1* gene differ by in-frame insertions of short GC-rich palindromic cluster (GC-cluster) in the coding region (Hudspeth et al., 1984). The terminal sequences of most GC clusters are repeats and different GC clusters can share the same terminal repeats. For instance, AG dinucleotide and TAG trinucleotide repeats are common among GC-clusters in *S. cerevisiae* (Weiller et al., 1989). The terminal repeats are regarded as target-site duplication, and GC-clusters have been suggested to bear ribozyme activity, which catalyzes self-cleavage and ligation reactions (Weiller et al., 1989; Lang et al., 2014). GC-cluster sequences rapidly accumulate substitutions especially in the loop regions, and also undergo dynamic merger and shuffling to form new GC-clusters (Wu and Hao, 2015). Changes at nucleotide bases as well as sequence structure result in highly variable GC cluster sequences among different yeast mitogenomes. GC-clusters are most often found in intergenic regions, but many of them are transcribed into RNAs (Wu and Hao, 2015). All these support the notion that GC clusters are transposable elements. GC-clusters can also be found in protein-coding regions. In yeast *Magnusiomyces capitatus* (in the Dipodascaceae family), GC-clusters inserted in protein-coding regions are transcribed in mRNAs, but the GC-cluster region in mRNA gets bypassed (or ignored) during translation (Lang et al., 2014). GC clusters have been suggested as recombination hotspots in mitogenomes (Dieckmann and Gandy, 1987). GC cluster–mediated gene conversion can insert or delete large genomic fragments (Weiller et al., 1991), which ultimately lead to alteration of genome size. GC clusters have been suggested to induce long AT-rich sequences into the *Nakaseomyces bacillisporus* mitogenome (Bouchier et al., 2009).

## Mitochondrial DNA Recombination

Pioneer studies on mtDNA recombination through mating between *S. cerevisiae* strains can be traced back to the early 1970s (Kleese et al., 1972; Shannon et al., 1972). Recent large-scale genomic survey confirms frequent mtDNA recombination in natural *S. cerevisiae* populations (De Chiara et al., 2020). Surprisingly, yeasts were not among the organisms in early discoveries of mtDNA recombination between different species, as shown in plants (Hao et al., 2010; Mower et al., 2010). Subsequent analysis on closely related yeast mitogenomes found extensive recombination throughout the mitogenome between yeast species (Wu et al., 2015). There are two important identified issues in assessing the extent of mtDNA recombination events. (1) Accurate detection of mtDNA recombination relies on the abundance of closely related mitogenomes, preferably, an abundant number of intraspecific mitogenomes from several related species. (2) Many mtDNA recombinant events are fine-scale and often overlooked when using whole genes as the unit of phylogenetic analysis.

Fritsch et al. (2014) have constructed the genome-wide map of mtDNA recombination events in *S. cerevisiae* and found that recombinant hotspots are preferentially localized in intergenic and intronic regions. They further investigated the impact of individual depletion of four genes [namely *Ntg1* (Ling et al., 2007), *Mgt1* (Lockshon et al., 1995), *MHR1*, and *Din7* (Ling et al., 2013)] previously associated with mtDNA recombination. The deletion of *Ntg1*, *Mgt1*, and *MHR1* had little influence on mtDNA recombination hotspots, and the deletion of *Din7* resulted primarily in DNA degradation. These findings hint that the study of only nuclear-encoded genes is unlikely to achieve a complete understanding of the localization of recombination hotspots along the yeast mitogenome. Given the dynamic nature of mitochondrial -encoded sequences, such as introns and GC-clusters, a plausible alternative could be that mtDNA recombination hotspots are primarily driven by the sequence features of the mitogenome. Future studies are needed to test this hypothesis.

Mitochondrial DNA recombination can impact mitochondrial function in different ways. As described above (on GC-clusters), Non-allelic mtDNA recombination can lead to deletion and insertion of sequences. Allelic mtDNA recombination, on the other hand, can prevent mtDNA deletion and repair mitogenome damage (Ling et al., 2019). Mitochondrial recombination introduces rapid sequence changes, and could have a significant functional impact on the host. There are direct functional impacts of the introduced mito-genotype, effects of the altered mito-nuclear interaction, and effects of the interaction between the introduced mito-loci and native mito-loci (mito-mito interaction) (Wolters et al., 2018). Mitochondrial recombination can enhance phenotypic variation among diploid hybrids, and facilitate the phenotypic differentiation of hybrid species (Leducq et al., 2017). Given the prevalence of yeast mtDNA recombination and even across different species, much phenotypic diversity in yeast could have resulted from mitochondrial recombination.

## Yeast Mitogenomes are Highly Variable in Size

**FIGURE 1:**
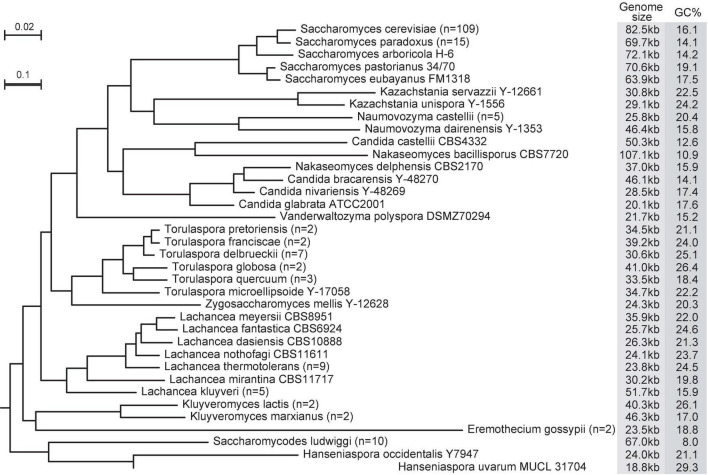
Variation in size, sequence divergence, and GC-content of mtDNAs of 36 yeast species. The phylogenetic tree is based on nucleotide sequences of seven core mitochondrial genes (*cob*, *cox1*, *cox2*, *cox3*, *atp6*, *atp8*, and *atp9*). The very long branch leading to *Hanseniaspora uvarum* is shown by a thickened line at fivefold reduced scale. If two or more strains are analyzed in a species, the averages are shown for genome size and GC% (strain number in parentheses). Otherwise, the strain names are shown. Details of all strain names in the Saccharomycetaceae family are in Xiao et al. (2017), and the *Saccharomycodes ludwigii* strains are in Nguyen et al. (2020b).

In the Saccharomycetaceae family, the smallest and largest mitogenomes belong in the same genus *Nakaseomyces* (Figure 1). The mitogenome in *Candida glabrata* is 20.1 kb (Koszul et al., 2003), while the mitogenome in *Nakaseomyces bacillisporus* is over five-time larger, at 107.1 Kb (Bouchier et al., 2009). In contrast, the sizes of nuclear genomes among the *Nakaseomyces* species are remarkably similar, ranging from 10.2 to 12.3 Mb (Gabaldon et al., 2013). In the sister family Saccharomycodaceae, *Hanseniaspora uvarum* has a linear mitogenome at 11.1 kb (Pramateftaki et al., 2006), and *Saccharomycodes ludwigii* has a circular mitogenome at 69.0 kb (Nguyen et al., 2020b). Mitogenome sizes also vary among strains within the same yeast species. Among the 109 *S. cerevisiae* mitogenomes examined in Xiao et al. (2017), and their genome sizes range from 74.2 to 92.2 kb, The fast increasing number of yeast mitogenomes will only expand the range in mitogenome size difference.

## Stable Gene Content in Yeast Mitogenomes

Despite the variable sizes, genes encoded in yeast mitogenomes are remarkably stable. Unlike mitogenomes in many other fungal species, none of the mitogenomes in Saccharomycetaceae and Saccharomycodaceae encode the respiratory-chain NADH dehydrogenase (complex I) (Dujon, 2010; Freel et al., 2014). The loss of complex I in yeasts is generally believed as a result of adaptation to fermentative lifestyles, where complex I is not essential (Schikora-Tamarit et al., 2021). All complete mitogenomes in Saccharomycetaceae encode eight protein genes, seven respiratory-chain protein genes (*atp6*, *atp8*, *atp9*, *cob*, *cox1*, *cox2*, and *cox3*) and one ribosomal protein gene *var1*, SSU and LSU rRNAs, and 22–24 tRNAs. This is in contrast with a recent report that “no gene is universally conserved in fungal mitogenomes” (Fonseca et al., 2021). Although it is inevitable that fewer mitochondrial genes are shared when more diverse lineages are included (Roger et al., 2017), the variation of mitogenome sequence quality could have been an important reason for the discrepancy on gene conservation among yeast mitogenomes. For instance, the two *S. cerevisiae* mitogenomes (accessions: CM002421 and CP046458) reportedly missing *atp6* by Fonseca et al. (2021) are 22,149 bp (including 13,247 Ns for gaps) and 49,451 bp (517 Ns) in length, respectively, much shorter than the average 82.5 Kb [ranging from 74.2 to 92.2 kb in Xiao et al. (2017)] of *S. cerevisiae* mitogenomes (Figure 1). The Saccharomycodaceae family has a sole case of gene loss involving a protein gene. That is the *var1* gene absent from *Hanseniaspora uvarum* (Pramateftaki et al., 2006). This is likely a lineage-specific gene loss, as *var1* is present in *Saccharomycodes ludwigii* (Nguyen et al., 2020b), a related species within the Saccharomycodaceae family.

## Factors Driving Mitogenome Size Variation

Mobile introns and variable intergenic regions are known factors driving variation of yeast mitogenome architecture (Bouchier et al., 2009; Freel et al., 2014). Ironically, the mitogenome in *Nakaseomyces bacillisporus* at 107 kb contains no intron, while mitogenomes in *Candida glabrata* at around 20 kb contain 3–4 introns in at least four intron-distribution patterns [(Koszul et al., 2003), and unpublished observation]. This irony can be solved by separate analyses at different time scales (Xiao et al., 2017). When intraspecific mitogenomes (within the same species) are compared, intron sequences show the highest variance in length and significantly overrepresented in large mitogenomes. When interspecific mitogenomes (among different species) are compared, tandem repeats show the highest variance in sequence length and significantly overrepresented in large mitogenomes. In other words, the rapid turnover of mobile introns can significantly impact genome size, but the number of available introns insertion sites are limited; while expansion and contraction of repeats may cause only subtle change per event, but they take place persistently with little space limit.

## Origin of Mitogenome Size

The question on whether genome size is under selective constraint has been a subject of debate (Lynch and Conery, 2003; Whitney and Garland, 2010; Shtolz and Mishmar, 2019). The mutational burden hypothesis (MBH) was postulated to explain the origin of organellar genome size (Lynch et al., 2006). Introns and intergenic DNAs in mitogenomes are genetic liability, as they are targets for deleterious and potentially lethal mutations (Lynch et al., 2006). Following MBH, introns and intergenic DNAs tend to accumulate when natural selection is less efficient at purging hazardous non-coding DNA. Using the ratio of non-synonymous over synonymous Ka/Ks rates as a proxy for the level of genetic drift, a significant positive correlation was evident between the genome-wide Ka/Ks ratios and mitogenome sizes among seven yeast species with sufficient intraspecific diversity (Xiao et al., 2017). This finding is consistent with the notion that introns, GC-clusters, and repeats in yeast mitogenomes are mostly deleterious (Bernardi, 2005; Wu and Hao, 2014, 2015). *Hanseniaspora uvarum* has accelerated sequence evolution compared with related species (Figure 1) and has a small genome size, which is consistent with the MBH hypothesis. Genetic drift still faces challenges to explain mitogenome size variation in many yeast species. For example, significant relaxation of mitochondrial functions has been documented after whole-genome duplication (Jiang et al., 2008), yet many post-whole-genome duplication species (including *Candida glabrata*) have compact mitogenomes. Unfortunately, many yeast species either lack intraspecific genomic data or suffer insufficient sequence diversity, making it impossible to estimate the degree of genetic drift. To address this issue, extended efforts are needed to sample and sequence an abundant number of intraspecific strains in a broad range of yeast species.

## Extreme Genome-Wide G + C Content

Even though yeast mitogenomes are overall AT-rich, their G + C-contents vary greatly. GC-content of the *Hanseniaspora uvarum* mitogenome is 29.3% (Pramateftaki et al., 2006), while the most AT-rich mitogenome in *Saccharomycodes ludwigii* is at 7.6% G + C (Nguyen et al., 2020b). Generally speaking, mutation is nearly universally biased from C/G to T/A (Hershberg and Petrov, 2010), and mutation rates are often higher at C/G nucleotides than at A/T nucleotides (Zhu et al., 2014). Alterations of GC content have been shown to impact mutation and recombination rates (Kiktev et al., 2018). The mitogenomes with extreme base composition offer important and unique insights into the mechanisms governing mutation processes (Gardner et al., 2002; McCutcheon and Moran, 2010; Smith et al., 2011; Su et al., 2019). Comparative genomics of the 10 extreme AT-rich mitogenomes in *Saccharomycodes ludwigii* (Nguyen et al., 2020b) found a strong mutation bias toward A/T, but the expected equilibrium G + C content under mutation pressure alone is still higher than observed G + C content. Interestingly, mitogenomes in *Saccharomycodes ludwigii* undergo frequent recombination, a genetic process that normally increases G + C content by GC-biased gene conversion (Pessia et al., 2012). These findings suggest other mechanisms alongside with AT-biased mutation operating to increase A/T in *Saccharomycodes ludwigii*. Another important, but perhaps underappreciated finding is the prevalence of indel mutations in yeast mitogenomes (Xiao et al., 2017). Indel mutations contribute much more to genomic variation among closely related mitogenomes than nucleotide substitutions. Further studies are needed to investigate the molecular mechanisms driving indel mutations and quantitatively model the evolutionary process of indel mutations.

## Variable Evolutionary Rates Among Yeast Mitogenomes

Yeast mitogenomes show variable evolutionary rates. The branch leading to *Hanseniaspora uvarum* is at least five times longer than the branch leading to its sister species. Similarly, the branch leading to a plant pathogen *Eremothecium gossypii* is at least three times longer than the branch leading to the related *Kluyveromyces* species (Figure 1). Spontaneous mitochondrial mutation rates have been measured in several yeast species. The mitochondrial base-substitution mutation (BSM) rates are all higher than their corresponding nuclear BSM rates. The mitochondrial BSM rates in *S. cerevisiae* range from 4.47 × 10^–10^ (Sharp et al., 2018) to 122.3 × 10^–10^ per site per cell division (Lynch et al., 2008). Please also note that the *S. cerevisiae* FY10 strain (isogenic to S288c) used in the Lynch et al. (2008) study contains a single non-synonymous mutation in the mtDNA polymerase (*MIP1*) linked to reduced fidelity of mtDNA replication (Baruffini et al., 2007b). *Hanseniaspora uvarum* does have a higher mitochondrial BSM rate than other two *Hanseniaspora* species, at 13.1 × 10^–10^ mutations per site per cell division, compared with 5.94 × 10^–10^ mutations per site per cell division in *Hanseniaspora valbyensis*, and 3.65 × 10^–10^ mutations per site per cell division in *Hanseniaspora osmophila* (Nguyen et al., 2020a). The measured spontaneous mutation rates will also help us estimate the effective population size for each species following equation π_*silent*_ = 2N_*e*_ μ, where π_*silent*_ is nucleotide diversity at silent sites, N_*e*_ is effective population size, μ is mutation rate (Lynch, 2006). Precise estimation of effective population size for mtDNA holds the key to understanding the significance of mtDNA recombination at the population level. To achieve this, it is critical to obtain both intraspecific diversity and spontaneous mutation rate for a variety of yeast species. Extra attention must also be paid to accumulate sufficient number of mitochondrial mutations due to the small mitogenome size relative to the nuclear genome size.

## Moving Forward

Yeast mitogenomes are highly diverse ranging from fast-evolving compact mitogenomes (similar to animal mitogenomes) to slow-evolving mitogenomes inflated by large introns, repeats and non-coding sequences (similar to plant mitogenomes). The fast-growing yeast mitogenome data have allowed us to begin to identify mechanisms driving genome diversity. Specific efforts are needed to sequence and study an abundant number of intraspecific strains from closely related species for contrast genomic features. The future of yeast mitogenome studies is bright, and the generated knowledge will no doubt benefit our understanding of mitogenomes much beyond the fungal kingdom.

## Author Contributions

WH wrote the manuscript.

## Conflict of Interest

The author declares that the research was conducted in the absence of any commercial or financial relationships that could be construed as a potential conflict of interest.

## Publisher’s Note

All claims expressed in this article are solely those of the authors and do not necessarily represent those of their affiliated organizations, or those of the publisher, the editors and the reviewers. Any product that may be evaluated in this article, or claim that may be made by its manufacturer, is not guaranteed or endorsed by the publisher.
